# Effect of lactase on symptoms and hydrogen breath levels in lactose intolerance: A crossover placebo‐controlled study

**DOI:** 10.1002/jgh3.12463

**Published:** 2020-12-01

**Authors:** Rajiv Baijal, Rakesh K Tandon

**Affiliations:** ^1^ Institute of Gastroenterology Pushpawati Singhania Hospital and Research Institute New Delhi India

**Keywords:** lactase enzyme, lactose hydrogen breath test, lactose intolerance

## Abstract

**Background and Aim:**

The absence of lactase in the intestinal villi due to mucosal injury or genetic factors causes undigested lactose to reach the colon where it is fermented. Lactose intolerance is diagnosed based on clinical symptoms like bloating, abdominal pain and flatulence, lactose hydrogen breath test (HBT), and lactose tolerance test. No Indian studies are available on the use of lactase supplements. The aim was to study the effect of lactase chewable tablets on clinical symptoms and hydrogen breath excretion in patients with lactose intolerance.

**Methods:**

This was a randomized, double‐blind, crossover placebo‐controlled trial to study the effect of lactase tablets on symptoms and hydrogen breath levels in adults with lactose intolerance, confirmed by Lactose HBT. Clinical symptom severity was recorded using a visual analog scale, and HBT was performed every 30 min for 180 min. As it was a crossover design, the same patients were tested with both lactase and placebo, acting as their own controls with a washout period of 1 week between visits.

**Results:**

Forty‐seven patients (mean age 33.6 years; 30 males) with lactose intolerance formed the study group. Clinical symptoms, mean clinical score (*P* < 0.05), and mean hydrogen breath levels (*P* < 0.05) were improved when the patients were given lactase. Reduction in cumulative hydrogen breath level over 180 min was 55% when patients received lactase compared to placebo.

**Conclusions:**

Orally supplemented lactase enzyme significantly reduced the clinical symptoms and hydrogen breath excretion in patients with lactose intolerance.

## Graphical abstract

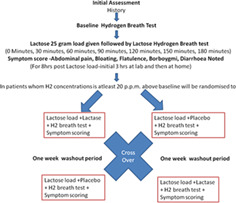

A randomized, double‐blind crossover placebo‐controlled trial to study the effect of Lactase tablets on symptoms and hydrogen breath levels in adults with lactose intolerance, confirmed by lactose hydrogen breath test. Orally supplemented lactase enzyme significantly reduced the clinical symptoms and hydrogen breath excretion in patients with lactose intolerance. Forty‐seven patients of lactose intolerance formed the study group. Orally supplemented lactase enzyme significantly reduced the clinical symptoms and hydrogen breath excretion in patients with lactose intolerance.

## Introduction

Lactase in the small intestinal villi breaks down lactose into glucose and galactose. In the absence of lactase due to mucosal injury or, more commonly, due to reduced genetic expression of the enzyme lactase‐phlorizin hydrolase,^1^ undigested lactose reaches the colon, where bacteria ferment it, generating H_2_, CO_2,_ lactic acid, and acetic acid, drawing water into the colon by osmosis. This results in clinical symptoms like diarrhea, abdominal pain, bloating, borborygmi, nausea, and flatulence after ingestion of milk or dairy products.

Often, at birth, there is insufficient lactase available in the intestinal villi. By 5–6 months, sufficient lactase starts being produced. The production of lactase enzyme starts decreasing after the age of 5–8 years.^2^

It is suggested that only about 35% of people worldwide are lactase‐persistent, who continue to produce lactase throughout adulthood, and thus are able to digest the sugar in milk without discomfort.^3^ Lactase deficiency can be seen in up to 15% of persons of Northern European descent, up to 80% of Blacks and Latinos, and up to 100% of American Indians and Asians.^4^ An Indian study conducted by Babu *et al*. indicated that the frequency and degree of lactose malabsorption is higher in South Indians compared to North Indians because of genetic differences in the population. The reported incidence was the highest in the south at 82% and 66% in the north.^5^

Tandon *et al*. suggested that the lower incidence of lactose intolerance in North Indian subjects may perhaps be because they are descendants of the Aryans who have been dairying for long and are known to be lactose tolerant.^6^

It is a common practice for clinicians to advise lactose‐intolerant patients to avoid milk and dairy products, but that may cause calcium, vitamin D, and riboflavin deficiency states; decrease quality of life; and lead to osteoporosis and depression. Lactase‐pretreated food products and external lactase enzyme are two of the dietetic treatments for lactose‐intolerant patients.^1^

It is therefore logical to coadminister lactase enzyme along with milk/dairy products so that the latter are digested and assimilated and yet do not produce the symptoms of intolerance.

The present study aimed to test this hypothesis. It was a randomized, double‐blind, placebo‐controlled trial to observe the effect of marketed lactase supplement on patients with lactose intolerance. The source of this lactase is *Aspergillus oryzae*, which is presently marketed in India as a food supplement. Although there are numerous international trials available, no trials on lactase enzyme have been conducted in India despite the supplement being used by many.

The aim was to study the effect of lactase chewable tablets (Yamoo) on clinical symptoms and hydrogen breath excretion in patients with lactose malabsorption and intolerance.

## Methods

The study was a randomized, crossover, double‐blind, placebo‐controlled trial, in which we studied the effect of an orally administered marketed lactase supplement (Lactase 4500 FCC chewable tablets) on hydrogen breath levels and intestinal symptoms in patients with lactose intolerance.

The trial was registered at Clinical Trials Registry India (ICMR‐NIMS) with registration number: CTRI/2018/03/012295.

The study was conducted at the Department of Gastroenterology, Pushpawati Singhania Research Institute for Liver, Renal & Digestive Diseases, New Delhi, in accordance with the ethical principles outlined in the Declaration of Helsinki, International Conference on Harmonization (ICH) Good Clinical Practice Guidelines, and local regulatory requirements.

An earlier study had shown that the hydrogen breath level was reduced from 31.8 ± 8.3 ppm to 14.7 ± 8.0 ppm on treatment with lactase (a decrease of 17.1 ppm). The placebo arm had shown a slight increase from 30.7 ± 8.7 ppm to 31.9 ± 8.0 ppm. An effect size of 0.5 was fixed under the assumption that similar results would be obtained for the present study. With statistical significance of 5%, a minimum study power of 80% and a patient dropout rate of 2%, each group would have 40 subjects. The study would have two arms—the experimental arm and the placebo arm—into which the patients would be randomly divided. To improve the statistical validity of the study, a crossover design would be used. Thus, we conducted a crossover, double‐blind, placebo‐controlled study with 50 patients.

Patients were randomized into two groups—A and B—such that half of them received treatment, and the other half received placebo, coded as Alpha and Beta, respectively. The randomization sequence was generated in advance by a computerized random number generator. Lactase and placebo tablets were identical in shape, size, color, taste, and smell. After a washout period of 1 week, the treatment assigned to each group was interchanged. We decided on an interceding period of 1 week to remove any residual effects of the previous treatment. All patients received both therapies, that is, lactase and placebo. Participants, investigators, and outcome assessors were blinded to the treatment assigned. The code was stored in a sealed envelope and retained under lock and was broken and disclosed to the investigators only after complete data entry was performed.

The study population consisted of patients between 18 and 65 years of age with a medical history consistent with lactose intolerance, confirmed by the lactose hydrogen breath test (HBT).

Patients were included if they were able to comprehend the study instructions, complete the requisite visits, consensually participate in the study, and sign the informed consent form (ICF). We included patients from Delhi and NCR (National Capital Region) because of the need for a minimum of three visits, 1 week apart.

Patients were excluded from the study group if they had diverticular disease, diabetic gastroparesis, HIV, immunosuppression of any origin, or cancer under treatment. Patients with conditions that could compromise participation in the study—such as hypersensitivity or previous laboratory or clinical adverse events related to the use of lactase or any of the components of the formulations used in the study, systemic infection during the study or use of antibiotics in the last 4 weeks, diabetes mellitus, uncontrolled hypertension or renal failure, pregnancy, lactation, and chronic pulmonary disease—were also excluded.

The study duration was 1 year, conducted from March 2018 to April 2019.

At the first visit, patients with a history of lactose intolerance fulfilling the inclusion criteria were clinically assessed.

A baseline HBT using the Bedfont Gastrolyzer hand‐held breath analyzer was performed. To minimize basal hydrogen excretion, patients were asked to have a carbohydrate‐restricted dinner on the day before the test and fast for at least 12 h on the day of the test. Before starting the test, patients washed their mouths with 20 ml of 0.05% chlorhexidine. Smoking and physical exercise was not allowed for 30 min before and during the test. End‐alveolar breath samples were collected immediately before lactose ingestion, and then, a dose of 25 g of lactose was administered, and breath samples were collected every 30 min for 3 h (0, 30, 60, 90, 120, 150, and 180 min). Symptom scores for abdominal pain, bloating, flatulence, borborygmi, and diarrhea were recorded at these intervals using the visual analog scale.

The test was considered positive (lactose malabsorption) if hydrogen concentrations were at least 20 ppm above baseline.

Patients were randomly allocated to two groups—A and B. Each patient was called a week later. At the second visit, a similar procedure was repeated, but this time, the patients in group A consumed three chewable lactase 4500 FCC tablets 5 min before the 25‐g lactose load, while patients in group B were given a placebo. The HBT values and symptoms were recorded as previously described. One week later, at the third visit, patients of group A received placebo, while group B received lactase tablets. Hence, each patient from each group was exposed to the experimental product and the placebo, with a washout interval of 1 week.

**Figure 1:**
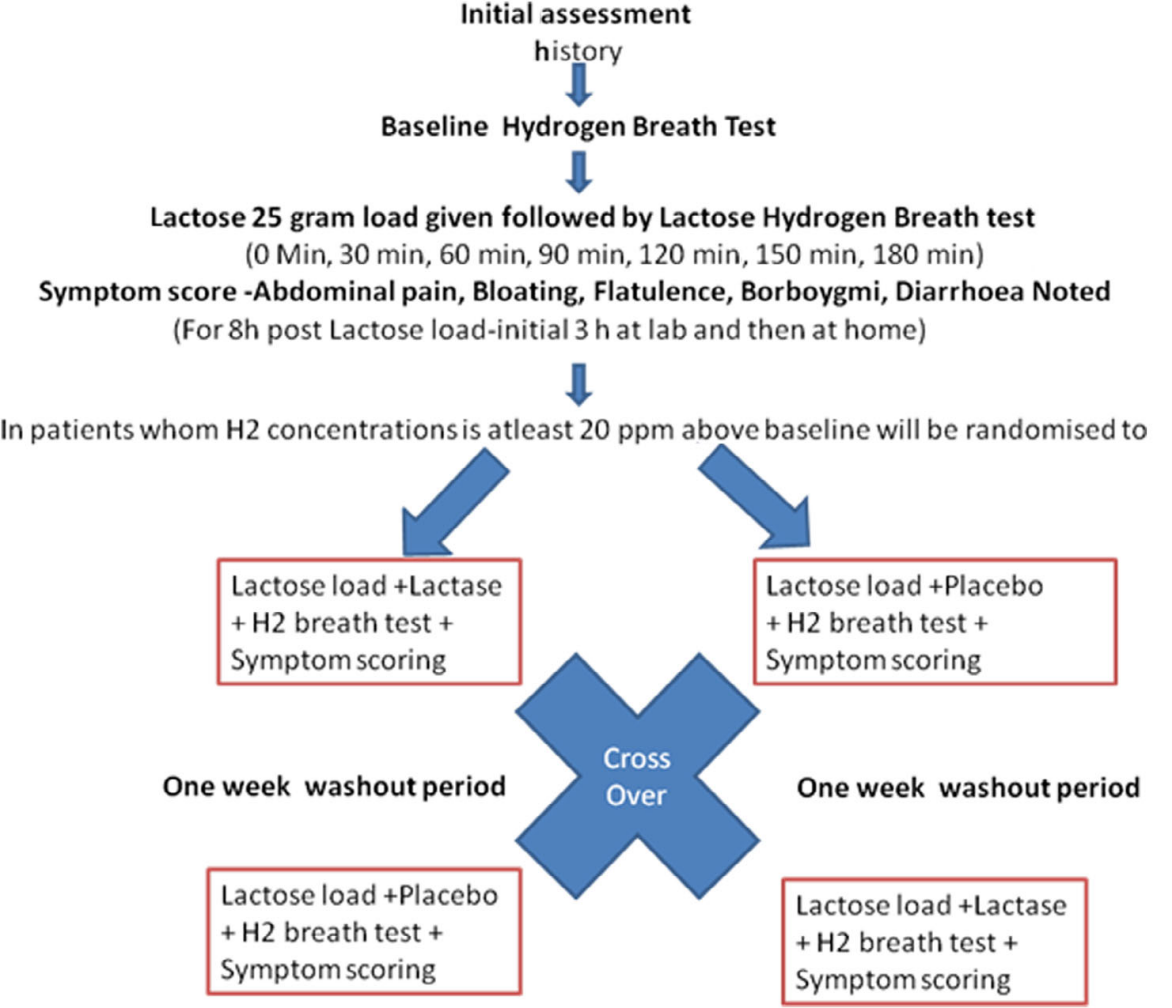
Flow chart showing the trial methodology.

The study design is illustrated in Figure 1.

Adverse events were monitored throughout the study.

For each group, mean clinical score of symptoms and mean hydrogen levels (ppm) at each time interval for each visit were calculated.

### *Statistical analysis*

Categorical variables were presented in number and percentage (%), and continuous variables were presented as mean. Normality of data was tested using the Kolmogorov–Smirnov test. If the normality was rejected, then a nonparametric test was used. Quantitative variables were compared using the Mann–Whitney Test (as the datasets were not normally distributed) between the two groups. A *P*‐value of <0.05 was considered statistically significant. The data was entered into an MS Excel spreadsheet, and analysis was carried out using Statistical Package for Social Sciences (SPSS) IBM version 21.0.

Area under the curve for cumulative hydrogen breath excretion over 180 min was calculated and compared between the placebo–lactase crossover groups (*n* = 47) using the trapezoid rule in MS Excel.

In order to account for errors in random sampling, the margin of error for difference of means in the HBT levels and overall symptom levels (expressed as the sum of each of the levels of abdominal pain, bloating, flatulence, borborygmi, and diarrhea using the visual analog scale) for the placebo and lactase groups was determined as the product of standard deviation and *z*‐value at 95% and 99% confidence intervals each.

## Results

Sixty‐seven patients were tested for lactose intolerance by HBT, of which 50 were found to have lactose intolerance. Three patients were lost to follow‐up; hence, 47 (mean age 33.6 years; 30 males, 17 females) patients formed the study group. These were randomized into two groups, group A (*n* = 23) and group B (*n* = 24).

No significant adverse events were detected throughout the study.

There was no statistically significant difference in clinical symptoms and mean clinical score (abdominal pain, bloating, flatulence, borborygmi, diarrhea) and hydrogen breath levels between groups A and B at baseline (Table 1).

**Table 1:** Baseline mean clinical score and hydrogen breath values at visit 1

	Baseline mean clinical score			Baseline mean hydrogen breath levels (ppm)		
Time (minutes)	Group A (*n* = 23)	Group B (*n* = 24)	*P* value	Group A (*n* = 23)	Group B (*n* = 24)	*P* value
0	1.516	1.836	0.248	39.70	38.54	0.840
30	1.666	1.764	0.308	45.56	45.38	0.758
60	1.798	1.900	0.354	93.35	76.83	0.544
90	1.926	1.544	0.446	113.74	89.83	0.400
120	1.694	1.400	0.476	132.44	105.00	0.202
150	1.524	1.428	0.550	136.61	130.83	0.873
180	1.476	1.390	0.498	146.22	140.83	0.655

At visit 2, when group A received lactase and group B received placebo, clinical symptoms and mean clinical scores were lower in group A than in group B, and hydrogen breath levels were lower in group A compared to group B, and the difference was statistically significant at 60, 90, 120, 150, and 180 min (Table 2).

**Table 2:** Mean clinical score and hydrogen breath values at visit 2 when group A received lactase and group B received placebo

	Mean clinical score			Mean hydrogen breath levels (ppm)		
Time (minutes)	Group A (*n* = 23) (lactase)	Group B (*n* = 24) (placebo)	*P* value	Group A (*n* = 23) (lactase)	Group B (*n* = 24) (placebo)	*P* value
0	0.262	1.458	0.253	25.83	31.67	0.183
30	0.484	1.700	0.319	28.17	33.33	0.079
60	0.662	1.508	0.171	39.83	53.08	0.027
90	0.392	1.474	0.199	53.52	78.92	0.012
120	0.234	1.392	0.068	56.96	96.38	0.021
150	0.348	1.302	0.267	59.78	98.42	0.012
180	0.244	1.132	0.266	58.44	109.46	0.008

At visit 3, when group B received lactase and group A received placebo, clinical symptoms and mean clinical score (abdominal pain, bloating, flatulence, borborygmi and diarrhea) were lower in group B than in group A. The difference between mean clinical score for groups B and A was statistically significant, with a *P*‐value of 0.041 at 120 min specifically for abdominal pain, bloating, and flatulence (Table 3).

**Table 3:** Mean clinical score and hydrogen breath values at visit 3 when group A received placebo and group B received lactase

	Mean clinical score			Mean hydrogen breath levels (ppm)		
Time (minutes)	Group A (*n* = 23) (placebo)	Group B (*n* = 24) (lactase)	*P* value	Group A (*n* = 23) (placebo)	Group B (*n* = 24) (lactase)	*P* value
0	1.148	0.268	0.293	24.39	29.75	0.966
30	1.268	0.400	0.279	27.70	26.96	0.348
60	1.380	0.368	0.246	56.44	39.04	0.198
90	1.068	0.158	0.257	82.56	43.83	0.019
120	1.010	0.056	0.041	85.17	48.92	0.009
150	0.766	0.048	0.255	94.91	56.33	0.022
180	0.688	0.056	0.117	95.52	57.88	0.022

At visit 3, when group B received lactase tablets, the hydrogen breath levels were lower compared to group A, and the difference was statistically significant at 90 min and subsequently till 180 min (Table 3).

**Figure 2:**
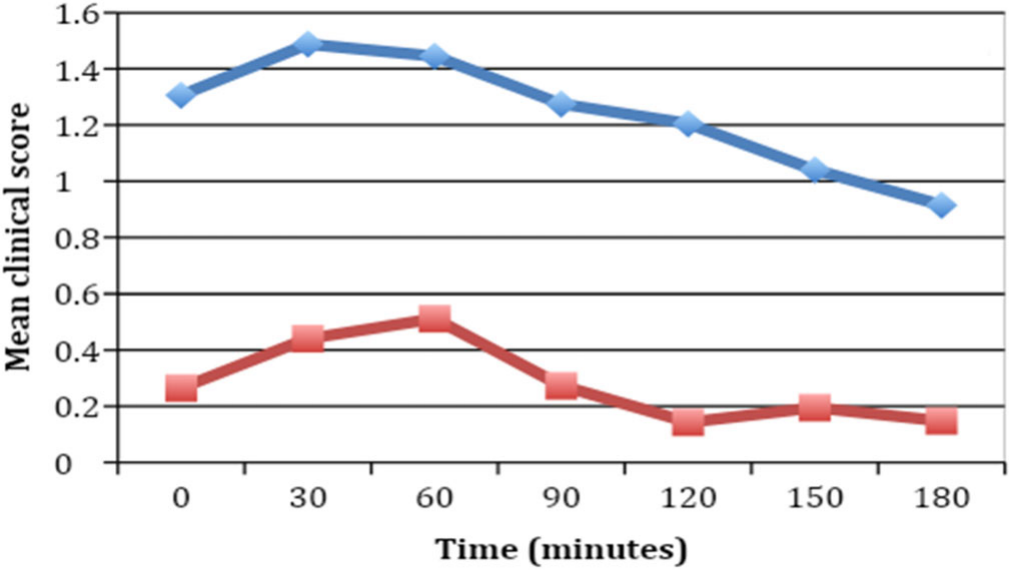
Comparison of mean clinical scores between lactase and placebo groups after crossover analysis (*n* = 47). 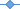, Placebo; 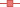, lactase.

**Figure 3:**
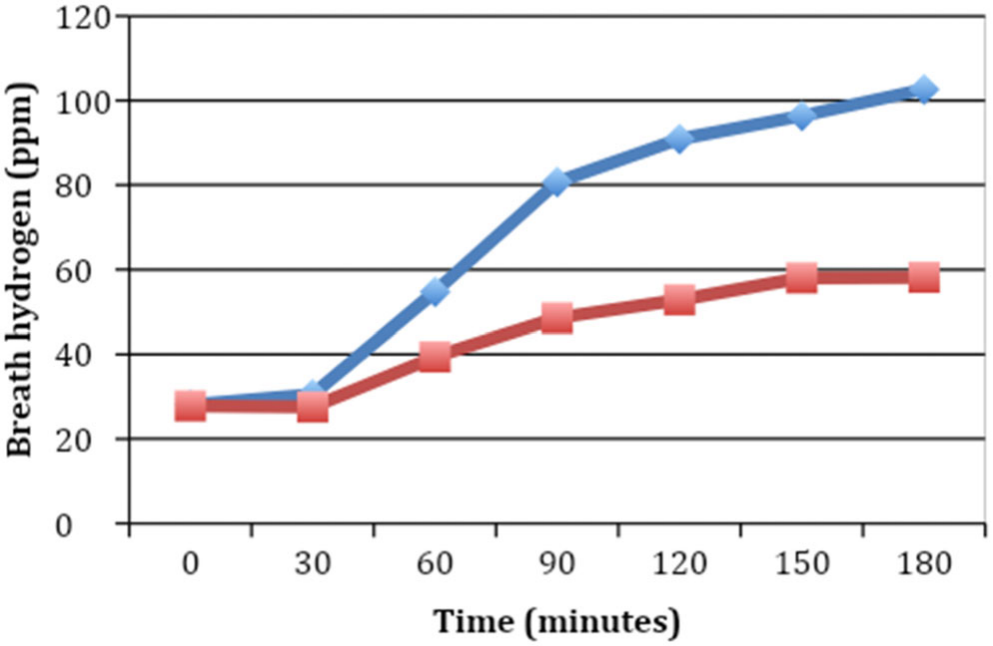
Comparison of hydrogen breath levels between lactase and placebo groups after crossover analysis (*n* = 47). 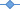, Placebo; 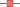, lactase.

In the crossover design, when a group of patients received lactase, the mean clinical score and the hydrogen breath levels were lower than when the same group of patients received placebo (Table 4, Figs 2, 3).

**Table 4:** Crossover design analysis of mean clinical scores and hydrogen breath levels (*n* = 47)

	Mean clinical score			Mean hydrogen breath levels (ppm)		
Time (minutes)	Placebo (*n* = 47)	Lactase (*n* = 47)	*P* value	Placebo (*n* = 47)	Lactase (*n* = 47)	*P* value
0	1.306 (0.90)	0.265(0.13)	0.029	28.11(8.22)	27.83 (6.69)	0.398
30	1.488(0.85)	0.441(0.10)	0.021	30.57(8.92)	27.55 (7.05)	0.346
60	1.445(0.81)	0.512(0.19)	0.033	54.72(16.00)	39.43(11.19)	0.116
90	1.275(0.82)	0.273(0.16)	0.024	80.70(23.58)	48.57(16.61)	0.033
120	1.205(0.70)	0.143(0.14)	0.005	90.89(26.50)	52.85(20.24)	0.029
150	1.040(0.72)	0.195(0.19)	0.032	96.35(28.10)	58.02(20.67)	0.035
180	0.915(0.65)	0.148(0.16)	0.030	102.64(29.93)	58.15(22.95)	0.025

All values in mean (standard deviation).

A difference in means of clinical symptom values that are lower than the margin of error is indicative of the action of the drug (Table S1, Supporting information). Margins of errors have been calculated at a 99% confidence interval. At time *t* = 0, the difference between means of the clinical symptoms expressed by patients in the placebo and lactase groups is 1.04, of which 0.36 is due to statistical error (with 99% confidence); the remaining is accounted for by the action of the drug. As time passes, the difference in means decreases monotonically along with the margins of error at a 99% confidence interval, indicating that, as time passes, the action of the drug accounts for a decrease in exhibited symptom.

As shown in Table S1, the difference of means for hydrogen breath levels that are lower than the margin of error is indicative of the action of drug rather than a difference due to sampling error. At 60 min, the margin of error at 99% confidence is 5.195 (confidence interval 10.10–20.49), indicating that the action of lactase leads to an observable decline in hydrogen breath levels.

Reduction in cumulative hydrogen breath level was 55% when patients received lactase compared to placebo. Maximum reduction took place in the final 30 min of the 3‐h period. The percentage reduction of hydrogen emitted in the breath monotonically increases with time, demonstrating that the efficacy of the drug increases over time (Table S2).

These results show that the clinical symptoms improved when patients received lactase compared to when they received placebo. The mean clinical score and mean and cumulative hydrogen breath values were significantly lower when the patients in either of the groups received lactase compared to placebo. As it was a crossover design, the same patients were tested with both lactase and placebo—indicating that clinical symptoms, mean clinical score, and mean and cumulative hydrogen breath levels were better whenever the patients were given lactase.

## Discussion

Our study showed that patients receiving lactase chewable tablets (Yamoo) had improvement in clinical symptoms of lactose intolerance after a lactose challenge. Their mean clinical score was better. Mean and cumulative hydrogen breath levels were also reduced when patients received lactase.

To ensure that any effects of the lactose intolerance test or lactase administration during visit 1 did not affect the results at visit 2, we set a gap of 1 week between the two visits.

The diagnosis of lactose intolerance can be based on clinical features, lactose tolerance test, and lactose HBT. Of them, the most reliable and convenient for the patient is indeed the lactose HBT that was used in the present study.

In most studies on lactose intolerance, the dosage of lactose has been either 25 g or 50 g. A 50‐g dose would likely have elicited a higher baseline score but is considered nonphysiological. Studies in India and elsewhere have found lower lactose doses to be acceptable for determining lactose malabsorption. Therefore, we have used 25 g of lactose in this study.

The administration of exogenous lactase as pills has been used to treat lactose intolerance in children, adolescents, and adults, and enzymatic supplementation is an intermediate step between dairy restriction and the use of diets with low levels of fermentable oligo‐, di‐, and monosaccharides and polyols (FODMAPs) for the management of lactose intolerance.^7^, ^8^

Lactase administration or pretreated food product with lactase enzyme is a practical therapeutic approach to control symptoms of lactase deficiency and lactose intolerance. Such treatment is also likely to prevent complications of reduced calcium intake and vitamin deficiency and to ensure a better quality of life.^7^

One of the limitations of this study is that the reduction in clinical symptoms does not correspond with the reduction in hydrogen breath values. This is likely due to the subjective nature of the clinical symptoms, as well as the Likert scale used for measuring them.

In all earlier trials, the HBT was used to diagnose excess hydrogen gas postlactose intake and postlactose intake with lactase. These trials showed reduction of hydrogen in breath of between 40% and 50%. Symptom score reduction was from 45% to 88%.^9^, ^10^, ^11^, ^12^

In conclusion, clinical symptoms like bloating, abdominal pain, diarrhea, flatulence, and borborygmi improved and hydrogen breath levels were reduced in lactose‐intolerant patients when they were given the lactase supplement.

## Supporting information


**Table S1** Variation of the impact of the drug with time as measured by difference in means, margin of error, and confidence intervals measured at 99% significance.

**Table S2** Comparison of total hydrogen breath levels between placebo and lactase groups as measured using trapezoid rule (*n* = 47).
