# Supplementary material for: Effect of lactase on symptoms and hydrogen breath levels in lactose intolerance: A crossover placebo‐controlled study
Source: JGH Open. 2020 Dec 1;5(1):143–8. doi: 10.1002/jgh3.12463 (PMC7812489; doi:10.1002/jgh3.12463)
Supplement: Supplementary file 1 — Table S1 Variation of the impact of the drug with time as measured by difference in means, margin of error, and confidence intervals measured at 99% significance. [file JGH3-5-143-s001.docx]

| **Table 5** Variation of the impact of the drug with time as measured by difference in means, margin of error and confidence intervals measured at 99% significance | | | | | | | | |
| --- | --- | --- | --- | --- | --- | --- | --- | --- |
| Time (minutes) | Clinical symptoms | | | | Hydrogen breath levels | | | |
|  | Difference in means (Placebo – Lactase) | Margin of error at 99% confidence (z=2.58) | Confidence interval at z = 2.58 (p = 0.005) | | Difference in means in ppm (Placebo – Lactase) | Margin of error at 99% confidence (z=2.58) | Confidence intervals at z = 2.58 (p = 0.005) | |
| 0 | 1.04 | 0.36 | 0.68 | 1.40 | 0.28 | 2.82 | -2.55 | 3.10 |
| 30 | 1.05 | 0.33 | 0.72 | 1.38 | 3.02 | 3.03 | 0.00 | 6.05 |
| 60 | 0.93 | 0.33 | 0.61 | 1.26 | 15.30 | 5.20 | 10.10 | 20.49 |
| 90 | 1.00 | 0.32 | 0.68 | 1.32 | 32.13 | 7.67 | 24.46 | 39.80 |
| 120 | 1.06 | 0.27 | 0.79 | 1.34 | 38.04 | 8.87 | 29.17 | 46.91 |
| 150 | 0.84 | 0.27 | 0.57 | 1.12 | 38.33 | 9.28 | 29.05 | 47.61 |
| 180 | 0.77 | 0.25 | 0.51 | 1.02 | 44.48 | 10.04 | 34.45 | 54.52 |
