# Supplementary material for: Effect of lactase on symptoms and hydrogen breath levels in lactose intolerance: A crossover placebo‐controlled study
Source: JGH Open. 2020 Dec 1;5(1):143–8. doi: 10.1002/jgh3.12463 (PMC7812489; doi:10.1002/jgh3.12463)
Supplement: Supplementary file 2 — Table S2 Comparison of total hydrogen breath levels between placebo and lactase groups as measured using trapezoid rule (n = 47). [file JGH3-5-143-s002.docx]

| **Table 6** Comparison of total hydrogen breath levels between placebo and lactase groups as measured using Trapezoid rule (n=47) | | | |
| --- | --- | --- | --- |
| Time Interval (minutes) | Breath Hydrogen levels (ppm minutes) | | % Reduction |
|  | With Placebo | With Lactase |  |
| 0 – 30 | 880 | 831 | 6 |
| 30 – 60 | 1279 | 1005 | 27 |
| 60 – 90 | 2031 | 1320 | 54 |
| 90 – 120 | 2574 | 1521 | 69 |
| 120 – 150 | 2809 | 1663 | 69 |
| 150 – 180 | 2985 | 1743 | 71 |
| Total breath hydrogen over 180 minutes | 12559 | 8082 | 55 |
